# The antifibrotic and fibrolytic properties of date fruit extract via modulation of genotoxicity, tissue-inhibitor of metalloproteinases and nuclear factor- kappa B pathway in a rat model of hepatotoxicity

**DOI:** 10.1186/s12906-016-1388-2

**Published:** 2016-10-24

**Authors:** Hala Attia, Nouf Al-Rasheed, Raeesa Mohamad, Nawal Al-Rasheed, Maha Al-Amin

**Affiliations:** 1 Department of Pharmacology and Toxicology, College of Pharmacy, King Saud University, Riyadh, 11495 Kingdom of Saudi Arabia; 2 Department of Biochemistry, College of Pharmacy, Mansoura University, 35516 Mansoura, Egypt; 3 Anatomy Department, Faculty of Medicine, King Saud University, Riyadh, 11495 Kingdom of Saudi Arabia

**Keywords:** Liver fibrosis, Date Fruits, Nuclear factor-kappa B, Cycloxygenase-2, Matrix metalloproteinase, 8-hydroxy deoxyguanosine

## Abstract

**Background:**

Hepatic fibrosis and its end point; cirrhosis, are the major cause of liver failure and death in patients with chronic liver disease. Therefore, the need for an effective treatment is evident. This study was designed to assess the potential effects of aqueous extract of date fruits, either flesh (DFE) or pits (DPE), on oxidative DNA damage and liver inflammation induced by carbon tetrachloride (CCl_4_) and whether they are related to inhibition of nuclear factor-κB pathway. In addition, the fibrolytic potential was evaluated via measuring matrix metalloproteinase-9 and tissue inhibitor of metalloproteinases −1 and −2.

**Methods:**

Rats were divided into the following groups: normal control, model control (CCl_4_ only), CCl_4_ + DFE, CCl_4_ + DPE and CCl_4_ + coffee. Coffee was used as a positive control. Fibrosis was induced by chronic administration of CCl_4_ (0.4 ml/kg) 3× a week for 8 weeks, and rats were treated with 6 ml/kg/day of DFE or DPE for 8 weeks. Liver homogenate was prepared for evaluation of oxidative stress, DNA damage, inflammatory and fibrolytic markers. Data are analyzed using one-way analysis of variance followed by a Tukey-Kramer post hoc test.

**Results:**

Both DFE and DPE significantly attenuated CCl_4_-induced oxidative damage as indicated by reducing lipid, protein and DNA oxidation in addition to increasing the levels of hepatic catalase activity. Both extracts blocked the accumulation of collagen I in the liver and ameliorated the increased expression of collagen III and α-smooth muscle actin suggesting suppression of profibrotic response induced by CCl_4_. DFE and DPE also upregulated the expression of heme oxygenase-1 and attenuated the nuclear factor-κB activation and cycloxygenase-2 expression reflecting their anti-inflammatory potential. Additionally, both flesh and pits extracts attenuated the increase in the tissue inhibitor of metalloproteinases −1 and −2 suggesting their fibrolytic activity.

**Conclusion:**

Our data suggest that DFE or DPE can prevent liver fibrosis by suppressing genotoxicity and nuclear factor-κB inflammatory pathway and by promoting collagen degradation.

**Electronic supplementary material:**

The online version of this article (doi:10.1186/s12906-016-1388-2) contains supplementary material, which is available to authorized users.

## Background

Hepatic fibrosis represents a wound-healing response of the liver to repeated injuries. It accompanied most chronic liver diseases caused by hepatitis viruses, alcohol abuse, metabolic or autoimmune diseases and chemical toxicity [1]. Cirrhosis is the end point of the fibrogenic process, and its complications- including portal hypertension, liver failure, and hepatocellular carcinoma- are the ultimate causes of death in most patients [2]. If treated properly, hepatic fibrosis can be reversed and its progression to irreversible cirrhosis may be prevented.

Liver fibrosis is characterized by the excessive synthesis and deposition of extracellular matrix (ECM) components, particularly collagens, resulting in derangement of the hepatic architecture by forming fibrous scars, and the subsequent development of nodules of regenerating hepatocytes. The major source of ECM accumulation following chronic liver injury is the transformation of hepatic stellate cells (HSCs) from quiescent vitamin A-storing cells into proliferative and fibrogenic myofibroblast-like phenotype, expressing alpha-smooth muscle actin (α-SMA), and dramatically increase the production of collagens [1, 2].

Matrix metalloproteinases (MMPs) are a group of zinc-dependent proteolytic enzymes that specifically promote ECM degradation [3]. Tissue inhibitors of metalloproteinases (TIMPs) are a group of functional peptides blocking ECM degradation by inhibiting MMPs proteolytic activity [4]. Under physiological conditions, MMPs and TIMPs are in homeostasis and they maintain hepatic ECM’s stability by regulating the formation and degradation. However, upon liver injury, an imbalance between MMPs and TIMPs activities has been developed and contribute to ECM accumulation and liver fibrosis [5]. Studies have shown that MMPs gene therapy can ameliorate experimental rat liver fibrosis [6, 7]. Furthermore, studies suggest that antibody and antisense oligonucleotides directed to TIMP-1 attenuate rat liver fibrosis [4]. Therefore, the interplay between MMPs and TIMPs provides a reference point to develop therapies for patients with ongoing fibrosis.

Collective evidences indicated that liver fibrosis incorporates uncontrolled inflammation as a part of its etiology. Nuclear factor- kappa B (NF-κB) is a transcription factor that serves as important regulators of the inflammatory response in liver fibrosis [8]. NF-κB consists of p65 and p50 subunits of Rel protein family and is ordinarily retained in the cytoplasm in an inactive form. Activation of NF-κB occurs after exposure of cells to oxidative stress and other physiological and pathological stimuli. Activated NF-κB translocates to the nucleus, where it regulates the expression of target genes involved in HSC activation, releasing of proinflammatory cytokines including tumor necrosis factor-alpha (TNF-α), Interleukin-6 (IL-6), IL-1β, and other inflammatory mediators such as cyclooxygenase-2 (COX-2) and inducible nitric oxide synthase (iNOS) which are involved in the process of fibrogenesis [9]. Some reports have indicated that hepatic injury induced by ischemia-reperfusion, alcohol, or endotoxin can be ameliorated by suppressing NF-κB activation in the liver [8, 10]. In addition, there is evidence showing that, competitive antagonism of NF-κB can inhibit the inflammatory response and prevent CCl_4_-induced hepatic injury and fibrosis [11]. Therefore, in the last few years, much attention has been focused on inhibiting NF-κB pathway as a target for prevention or treatment of liver fibrosis.

Chemical toxicity and chronic inflammation are tightly associated with the continuous generation of reactive oxygen species (ROS) which cause various types of tissue damage including lipid peroxidation, degradation of proteins, strand breakage of DNA, and formation of 8-hydroxy-2’- deoxyguanosine (8-OHdG) [12, 13]. Through these molecular derangements, highly concentrated ROS may give rise to parenchymal cell death, replaced by tissue fibrosis [14]. Furthermore, accumulation of 8-OHdG in DNA eventually brings about cellular mutagenesis and subsequent carcinogenesis. Because the liver is an iron-rich organ which contains 30 % of total body storage iron [15], it is considered to be one of the most susceptible organs to cellular damage or DNA mutagenesis caused by ROS. Therefore, prevention of oxidative genotoxicity is vital for inhibiting liver fibrosis and protection against hepatocarcinogenesis.

Heme oxygenase-1 (HO-1), an important antioxidant enzyme catalyzing the rate-limiting step in heme degradation, is one of the factors protecting against oxidative stress, inflammation, and metabolic dysregulation [16]. Recently, it has been reported that HO-1 induction by hemin protects the liver against obesity-induced metabolic dysregulation by reducing hepatic oxidative stress and reducing adipose inflammation [16]. In addition, up-regulation of HO-1 has been shown to protect liver cells from ischemia/reperfusion injury via antioxidant, anti-inflammatory and antiapoptotic mechanisms [17–19]. Formerly, Tsui et al. [20] suggested the induction of HO-1 in HSCs as a new approach for the treatment of liver fibrosis. Therefore and according to these findings, we hypothesized that dietary factors that promote HO-1 induction might help to protect liver from carbon tetrachloride (CCl_4_)-induced oxidative stress and inflammation.

Fruits of the date palm (*Phoenix dactylifera L*.) are very commonly consumed in many parts of the world and are a vital component of the diet and a staple food in the Middle East and in North Africa. Nutritional value of date palm fruit is attributed to its high contents of carbohydrates, salts, minerals, dietary fibers, vitamins, fatty acids, amino acids, and protein [21]. The date palm fruit possesses many useful properties such as antioxidant and antimutagenic activity [22–29], antibacterial [30], antifungal [31] and anti-tumoral [32]. Moreover, the aqueous and ethanolic extracts of dates, were effective in ameliorating the severity of gastric ulceration [33], nephrotoxicity [29] and neurotoxicity [34] via antioxidant mechanisms. Al-Qarawi et al. [35] and El-Gazzar et al. [25] demonstrated a hepatoprotective effect of date palm fruit extract on oxidative damage induced by CCl_4_. Moreover, aqueous extract of date fruit has a protective effect against thioacetamide-[24], trichloroacetic- [26] and dimethoate- [28] induced oxidative damage in rat liver. Date palm fruits are composed of a fleshy pericarp (flesh) and seeds (pits). The date pits is also high in polyphenolics and antioxidants [36]. The protective role of date pits extract on oxidative liver damage was also investigated [35, 37].

Our recent work [38] presented evidence that the antifibrotic effects of the aqueous extract of date flesh (DFE) and pits (DPE) are not exclusively via antioxidant mechanism but also through downregulation of inflammatory, fibrotic and angiogenic markers. In the present work we aimed to investigate whether the DFE or DPE could exert their antifibrotic effects via ameliorating the oxidative DNA damage and/or stimulating the fibrolytic processes (promotion of ECM degradation) through upregulation of MMPs and downregulation of TIMPs. In addition, our study aimed to explore whether the anti-inflammatory effects exerted by DFE and DPE in fibrotic liver are mediated through alleviation of NF- κB pathway and/or upregulation of HO-1 activity.

Coffee is one of the most popular and highly consumed beverages worldwide. Extensive studies demonstrated the beneficial role of coffee and its components in the protection from various liver diseases [39–42]. Moreover, experimental studies have suggested that the intake of instant coffee, conventional coffee, or any of its components can reduce hepatotoxin-induced liver fibrosis in rats and mice [43–48]. Coffee is a rich source of polyphenols and diterpenes which contribute to the ability of coffee to suppress the genotoxicity [49–51] and NF-κB pathway [52–54] and to enhance HO-1 activity [55, 56] in different animal models. Therefore and based on its potential and beneficial protective effects on liver, coffee was selected as a reference hepatoprotective agent in the current study.

## Methods

### Animals

This study was conducted on adult male albino Wistar rats weighing 180–200 g provided by the Experimental Animal Center of King Saud University, College of Pharmacy, Riyadh, KSA and housed at 21–25 °C and a 12 h light/dark cycle in a well-prepared animal house. The animals had free access to pellet food and tap water. The research was conducted in accordance with the National Institutes of Health Guide for Care and Use of Laboratory Animals and approved by the Animal Care Committee of King Saud University.

### Chemicals, kits and antibodies

CCl_4_, thiobarbituric acid (TBA), trichloroacetic acid (TCA), Bilirubin Assay Kit, normal and low melting agarose and ethidium bromide were purchased from Sigma-Aldrich chemical Co. (St Louis, MO, USA). Date fruits of *Mabroom* variety were obtained from Kingdom Dates Factory in Riyadh, Kingdom of Saudi Arabia. Date pits powder was purchased from a local company in Riyadh, Kingdom of Saudi Arabia. Instant coffee (Nescafe®,) was obtained from Nestlé (Cheongju, Korea). Commercial kits used for liver enzymes were purchased from Randox Laboratories Ltd. (CRUMLIN, CO. Antrim, UK). ELISA kits for assay of MMP-9, TIMP-1 were obtained from R&D Co. (Quantikine, R&D systems, Minneapolis, MN, USA). ELISA kit for the assay of 8-hydroxy deoxyguanosine (8-OH-dG) was purchased from Abnova Co. (CA, USA). Primary antibodies for detection of α- SMA, COX-2, NF-κB p65, collagen I and TIMP-2 were obtained from Santa Cruz (Santa Cruz Biotechnology, CA, USA). Primary antibodies for immunostaining of HO-1, collagen III and MMP-9 were purchased from Abcam (Cambridge, UK). Secondary antibody was obtained from Sigma-Aldrich. All other reagents were of analytical quality.

### Preparation of date flesh extract

The aqueous date extract was prepared according to the method of Al-Qarawi et al. [35]. The date flesh was manually separated from the pits (seeds). The flesh is then soaked in cold distilled water in a ratio 1:3 (g/ml) and kept for 48 h in a refrigerator (4 °C) with continuous stirring. The extract was then filtered and the aqueous supernatant was used.

### Preparation of date pits extract

The dried pit powder was purchased from a local company in Riyadh. The powder was soaked with water in a ratio 1: 10 (g/ml) under agitation and kept at 4 ° C for 48 h. After 48 h, the extract was filtered and the aqueous supernatant was used [38]. Aqueous extract was selected for both flesh and pits to get the advantages of all antioxidant contents because most of the antioxidants and active components in dates are extracted in water [23].

### Preparation of CCl_4_ and induction of liver fibrosis

CCl_4_ was prepared by dissolving in corn oil (40 % v/v). Liver fibrosis was induced by intraperitoneal injection of 0.4 ml/kg of CCl_4_ 3× weekly for 8 weeks.

### Experimental design

Animals were randomly divided into five groups of ten rats each as follows: Group I: Normal control with no treatment; Group II: Model control rats injected with CCl_4_ only (0.4 ml /kg; i.p.; 3× weekly for 8 weeks); Groups III and IV: rats injected with CCl_4_ 3 × weekly for 8 weeks and concomitantly treated with aqueous date flesh extract (DFE; 6 ml/kg) or aqueous date pits extract (DPE; 6 ml/kg); respectively by oral gavage daily for 8 weeks. Group V: is the positive control group composed of rats injected with CCl_4_ 3× weekly for 8 weeks and concomitantly treated with coffee (300 mg/kg, dissolved in hot water). The dose of both DFE and DPE (6 ml/kg) was selected based on a previous study performed on our lab using 4 doses for each extract (2, 4, 6 and 8 ml/kg) in CCl_4_-treated rats [38]. The end points were Masson trichome staining to detect collagen deposition. The doses 6 and 8 ml/kg showed the best and similar improvement in collagen deposition, so the dose 6 ml/kg was chosen to complete the study [38]. The dose of coffee was chosen considering four standard cups as the amount drinking daily.

### Preparation of serum and tissue homogenate

At the end of 8 weeks, rats were anesthetized and sacrificed by decapitation. Blood samples were collected, and divided into two parts; one part was allowed to coagulate, centrifuged at 3000 rpm for 15 min to separate serum. Sera were used for determination of liver enzymes activities and bilirubin levels. Second part was collected in EDTA-tubes, and centrifuged to separate plasma. Plasma samples were used to determine the levels of advanced oxidation protein products (AOPPs). The livers were removed and cleaned from excess blood with ice-cold normal saline. A part of each liver was fixed with 4 % formalin in phosphate buffered saline (PBS; pH 7.4) for at least 24 h and prepared for immunohistochemical detection of α-SMA, NF-κB p65, COX-2, HO-1, collagen I, collagen III, MMP-9 and TIMP-2. One gram of each liver was sampled and homogenized (20 % w/v) in cold PBS (NaCl 8 g/L, KCl 0.2 g/L, Na_2_HPO_4_ 144 g/L and KH_2_PO_4_ 0.24 g/L, pH 7.4) by using Ultra-Turax (IKA-USA) homogenizer. The resulting tissue homogenates were centrifuged at 3000 rpm at 4 °C for ten minutes. The collected supernatant was divided into aliquots and then kept at −80 °C till being used for the assay lipid peroxides, catalase, 8 OH-dG, MMP-9 and TIMP-1.

### Evaluation of liver function

Serum activities of liver enzymes, alanine aminotransferase (ALT) and aspartate aminotransferase (AST), and serum levels of total and direct (conjugated) bilirubin were measured with routine laboratory methods using commercially available kits according to manufacturer’s instructions.

### Assessment of oxidative stress markers

#### Determination of thiobarbituric acid reactive substances (TBRS, a marker of lipid peroxidation

Lipid peroxidation was determined by estimating the level of thiobarbituric acid reactive substances (TBARS) measured as malondialdehyde (MDA), according to the method of Ohkawa et al. [57]. MDA is an end product in the sequence of lipid peroxidation reactions and so was taken as an index for this process. Briefly, the reaction mixture (0.5 ml homogenate + 2.5 ml 20 % TCA +1.0 ml 0.6 % TBA) was heated for 30 min in a boiling water bath. The mixture was then cooled and centrifuged for 10 min at 4 °C. The absorbance of the developed pink-colored product was measured at 535 nm against a reagent blank. 1, 1, 3, 3-tetraethoxypropane, a form of MDA, was used as standard in this assay. The results were expressed as nmol of MDA/g of wet tissue.

#### Assay of advanced oxidation protein products (AOPPs), a marker of protein oxidation

The determination of plasma AOPPs was based on spectrophotometric detection according to Witko-Sarsat et al. [58]. Briefly, 200 μl of plasma (diluted 1:5 with PBS as a test), 200 μl of chloramine-T solution (0–100 μmol/L) for calibration and 200 μl of PBS as a blank were applied. A 10 μl of 1.16 M potassium iodide and 20 μl of acetic acid were added, and the absorbance at 340 nm was measured immediately. The concentration of AOPPs was expressed as μmol/l of chloramine-T equivalents.

#### Assay of catalase (CAT) as enzymatic antioxidant

CAT activity was estimated as the decomposition rate of hydrogen peroxide according to the method of Aebi [59]. In brief, 50 μl of liver homogenate was diluted with 5 ml of phosphate buffer, and then 2 ml of the diluted homogenate was mixed with 1 ml hydrogen peroxide. The absorbance was read after 15 and 30 s at 240 nm.

### Markers of DNA damage

#### Assay of 8-OHdG

The hepatic levels of 8-OHdG (an abundant form of oxidative DNA damage) were determined by enzyme-linked immunosorbent assay (ELISA) using rat immunoassay kits (Abnova Co). According to the manufacturer’s instructions, two freeze-thaw cycles of liver homogenate samples were performed to break cell membranes, then the homogenates were centrifugated at 5000xg for 5 min and the assay was performed on the supernatant as indicated by the instructions.

#### Comet DNA assay (marker for DNA fragmentation)

The standard procedure was originally described by Singh et al. [60] and modified by Hu et al. [61]. A small piece of liver samples was minced, suspended in 4 ml chilled homogenizing solution (pH 7.5) containing 0.075 M NaCl and 0.024 M Na_2_EDTA and then homogenized gently at 500 to 800 rpm in ice. 100 μl of liver homogenate was mixed with 600 μl of low-melting agarose (0.8 % in PBS). 100 μl of this mixture was spread on slides pre-coated with 300 μl of 0.6 % normal melting point agarose (NMP). After application of a third layer of 0.6 % NMP (300 μl), slides were immersed in ice-cold lysis buffer (0.045 M Trisborate- EDTA (TBE) buffer, pH 8.4) for 1 h at 4 °C. Slides were then removed from the lysing solution and placed for 20 min in a horizontal electrophoresis unit filled with an alkaline buffer (1 mM Na_2_EDTA and 300 mM NaOH, pH 13) to allow the unwinding of DNA. After the unwinding of DNA, electrophoresis was carried out under standard conditions (25 V, 300 mA, and distance between electrodes 30 cm) for 20 min at room temperature in the same alkaline solution (pH 13). Electrophoresis at high pH results in structures resembling comets, as observed by fluorescence microscopy; the intensity of the comet tail relative to the head reflects the number of DNA breaks. The slides were then neutralized by adding 0.4 M Tris-HCl buffer (pH 7.5), stained with ethidium bromide (20 μg/ml) at 4 °C, covered and stored in sealed boxes at 4 °C until image analysis. All preparation steps were performed under dimmed light to prevent additional DNA damage by UV. Images of 100 randomly selected cells were analyzed for each sample. DNA fragment migration patterns was evaluated with Leitz Orthoplan epifluorescence microscope (magnification 250×) equipped with an excitation filter of 515–560 nm and a barrier filter of 590 nm. The microscope was connected through a camera to a computer-based image analysis system (Comet Assay IV software, Perspective Instruments). Comets were randomly captured at a constant depth of the gel, avoiding the edges of the gel, occasional dead cells, and superimposed comets. DNA damage was measured as tail length (TL = distance of DNA migration from the centre of the body of the nuclear core), tail intensity (TI = % of DNA that migrated from the nuclear core to the tail) and tail moment. The tail moment (TMOM) was determined according to the formula: TMOM = DNA in tail as a % of total DNA x tail length (TL).

#### ELISA assay of MMP-9 and TIMP-1

The hepatic levels of MMP-9 (a collagen degrading enzyme) and TIMP-1 (a tissue inhibitor of collagen degradation) were determined by enzyme-linked immunosorbent assay using rat immunoassay kits (R&D Systems) according to the manufacturer’s instructions.

### Immunohistochemistry for detection of collagen I, collagen III, α-SMA, COX-2, NF-κB p65, HO-1, MMP-9 and TIMP-2

The following primary antibodies were used for immunostaining of paraffin sections of the liver: goat polyclonal anti-collagen I antibody (sc-25,974), mouse monoclonal anti-collagen III antibody (ab 82,354), mouse monoclonal anti-α-SMA (sc-130,617), goat polyclonal anti-COX-2 (sc-1747), mouse monoclonal anti-NF-κB p65 (sc-8008), rabbit polyclonal anti-HO-1 (ab85309), rabbit monoclonal anti-MMP-9 (ab76003) and goat polyclonal anti-TIMP-2 (sc-6835). Immunostaining was performed using Immuno Cruz ABC staining system from Santa Cruz. The procedure involved the following steps: endogenous peroxidase activity was inhibited by 3 % H_2_O_2_ in distilled water for 5 min, and then the sections were washed in Tris buffered saline (Sigma, T 5030–100 TAB, pH 7.6) for 10 min. Non-specific binding of antibodies was blocked by incubation with protein block for 5 min. Sections were incubated with the primary antibodies (diluted 1:100, except for anti- α–SMA which is diluted 1:200) for 1 h at room temperature. Sections were washed in Tris buffer for 3 times each for 3 min, and then incubated with biotinylatedantirabbit IgG for 30 min. This was followed by washing in Tris buffer for 3 times, each for 3 min. Peroxidase was detected with working solution of Diaminobenzedine substrate for 10 min. Finally sections were washed in distilled water for 10 min, nuclei were stained with Mayer’s hematoxylin and sections were mounted in DPX. For negative control sections, the same procedure was followed with omission of incubation in the primary antibody.

### Statistical analysis

The results are expressed as the mean ± SEM. Statistical comparisons between the groups were performed using one-way analysis of variance (ANOVA) followed by a Tukey-Kramer post hoc test. Statistical analysis was conducted using Prism GraphPad software version 4 (San Diego, California, USA). *P* values < 0.05 were considered statistically significant.

## Results

### Effect of DFE and DPE on liver functions in CCl_4_-intoxicated rats

**Fig. 1:**
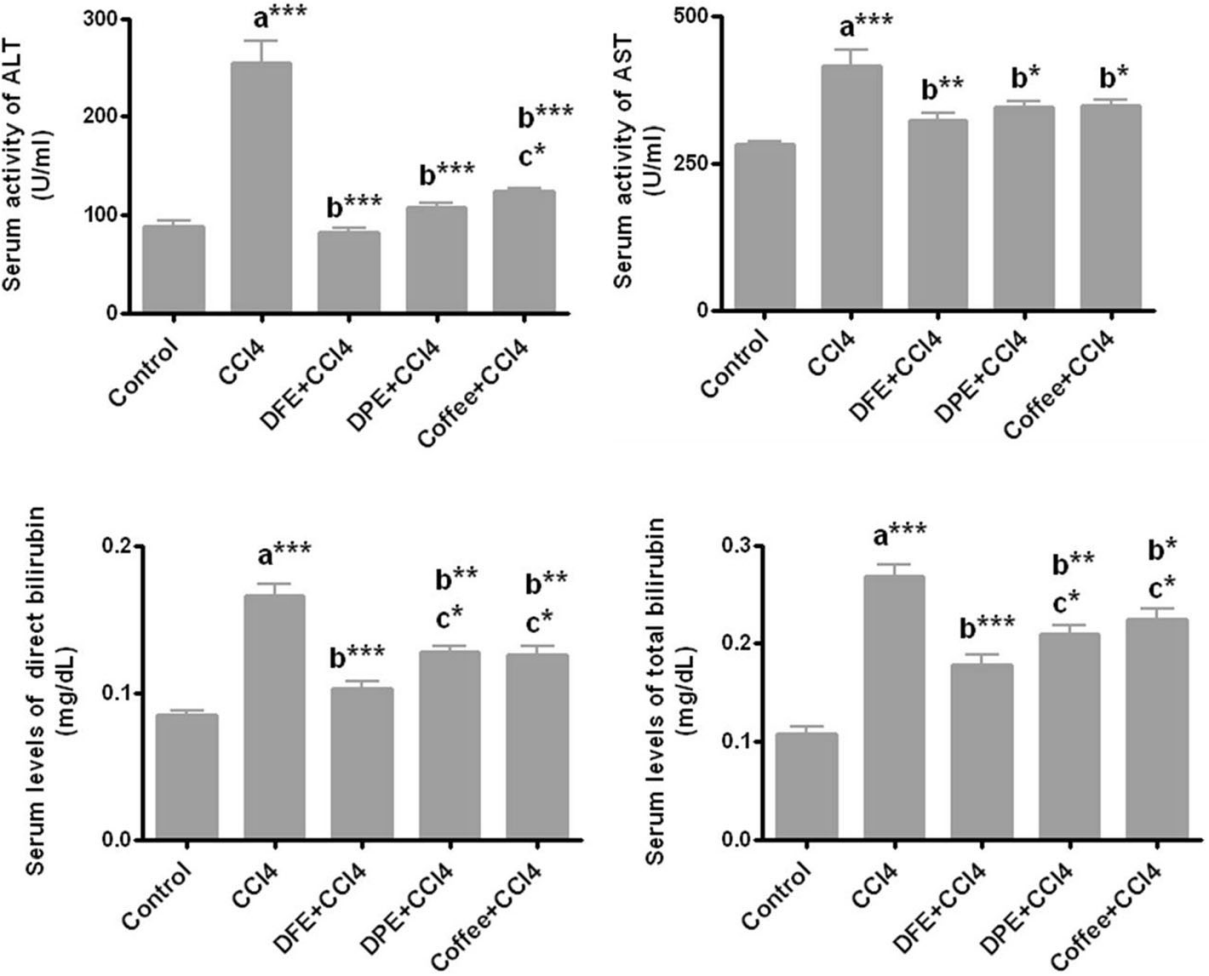
Effect of DFE, DPE and coffee on liver function markers in CCl_4_-intoxicated rats. **a** significantly different from normal control group; **b** significantly different from CCl_4_-treated group; **c** significantly different from DFE-treated rats. Values are expressed as mean ± SEM. *** *P* < 0.001, ** *P* < 0.01, * *P* < 0.05. ALT: alanine aminotransferase; AST: aspartate aminotransferase

Estimation of liver function revealed that CCl_4_ induced a significant increase in serum ALT and AST activities and in total and direct bilirubin levels compared to the normal control group (*P* <0.001). Serum levels of ALT, AST and both direct and total bilirubin were significantly reduced by the concurrent treatment with DFE, DPE and coffee compared to CCl_4_-intoxicated group (Fig. 1). DFE-treated rats showed significant lower levels of total and direct bilirubin compared to both DPE- and coffee-treated groups (*P* <0.05), and decreased ALT activity compared to coffee-treated rats (*P* <0.05).

### Effect of DFE and DPE on oxidative stress markers in CCl_4_-intoxicated rats

CCl_4_-induced oxidative stress in rat livers was evaluated by assessing lipid peroxides, AOPP and CAT (as an antioxidant enzyme) in hepatic tissues (Table 1). CCl_4_ significantly increased the levels of TBARS and AOPP compared to normal control group (*P* <0.001) reflecting both lipid and protein oxidation, respectively. Furthermore, CCl_4_ significantly reduced the hepatic activities of CAT compared to normal control group (*P* <0.001). Treatment with DFE, DPE or coffee concomitantly with CCl_4_ presented significant protection against CCl_4_-induced oxidative stress as indicated by the significant decrease in TBARS and AOPP levels (*P* <0.001) together with significant rise in the activities of CAT (*P* <0.001, *P* <0.01) compared to CCl_4_-intoxicated rats. DFE-treated rats showed significant improved levels of AOPP and CAT compared to both DPE- and coffee-treated groups (*P* <0.05), and lowered TBARS levels compared to coffee-treated rats (*P* <0.05).

**Table 1:** Effects of DFE, DPE and coffee on hepatic oxidative stress markers; TBARS (a marker of lipid peroxidation), AOPP (a marker of protein oxidation) and CAT (as enzymatic antioxidant) in CCl_4_-intoxicated rats. Values are expressed as mean ± SEM

	TBARS (nmol/g tissue)	AOPPs (μmol/L)	CAT (U/g tissue)
Control	72.4 ± 7.28	69.43 ± 0.44	6.74 ± 0.15
CCl_4_	158.7 ^a***^ ± 12.1	208.2 ^a***^ ± 10.7	4.23 ^a***^ ± 0.38
DFE+ CCl_4_	93.17 ^b***^ ± 5.62	114.1 ^b***^ ± 6.13	5.9 ^b***^ ± 0.147
DPE+ CCl_4_	107.8 ^b***^ ± 6.54	154.3^b*** c*^ ± 10.32	5.2 ^b**c*^ ± 0.2
Coffee + CCl_4_	129.3 ^b***c*^ ± 7.9	155.1 ^b*** c*^ ± 10.23	5.04 ^b**c*^ ± 0.26

***P < 0.001, **P < 0.01, *P < 0.05

^a^significantly different from normal control group; ^b^significantly different from CCl_4_-treated group, ^c^significantly different from DFE-treated rats

### Effect of DFE and DPE on markers of oxidative DNA damage in CCl_4_-intoxicated rats

**Fig. 2:**
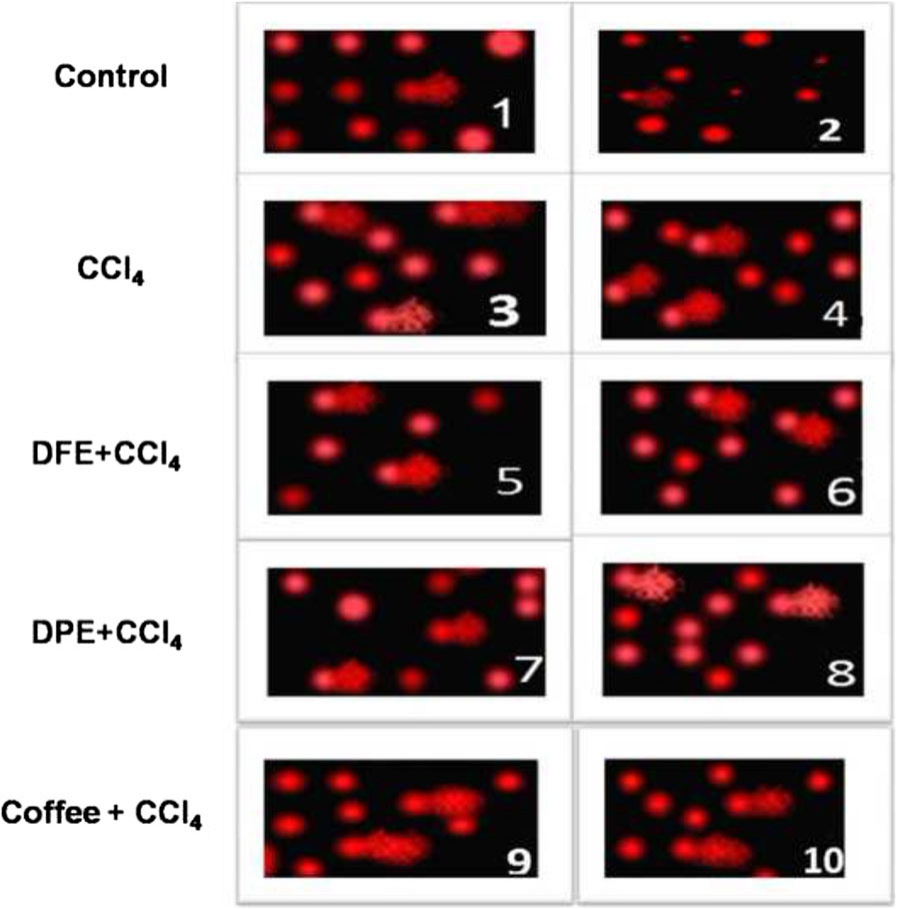
Effect of DFE, DPE and coffee on comet DNA assay in liver tissues in CCl_4_-intoxicated rats

**Fig. 3:**
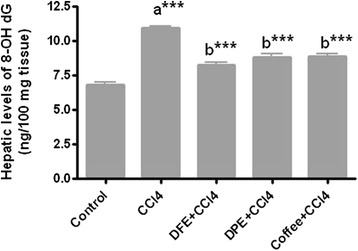
Effect of DFE, DPE and coffee on hepatic levels of 8-OHdG as a marker of DNA damage. Values are expressed as mean ± SEM. **a** significantly different from normal control group; **b** significantly different from CCl_4_-treated group. *** *P* < 0.001

Figures 2 and 3 & Table 2 demonstrated a DNA damage induced by CCl_4_ as assessed by measurement of 8-OHdG and comet assay. CCl_4_ resulted in marked elevation of 8-OHdG compared to normal control (*P* <0.001), however the concomitant treatment with DFE, DPE and coffee significantly subsided these levels compared to CCl_4_-intoxicated rats (*P* <0.001, Fig. 3). No significant difference was observed in DFE- or DPE- treated rats compared to coffee-treated group. Comet DNA assay (Fig. 2 and Table 2) indicated DNA damage in CCl_4_-intoxicated rats as revealed by significant increase in tailed DNA%, tail length, tail intensity and tail moment compared to normal control (*P* <0.001), while simultaneous treatment with DFE, DPE or coffee significantly diminished this damage (Table 2). Treatment with DFE significantly reduced the tail length (*P* <0. 05) and tail moment (*P* <0.001) compared to DPE, while no significant difference in any of DNA damage indices was observed compared to coffee-treated group

**Table 2:** The effects of DFE, DPE and coffee on comet DNA assay parameters in CCl_4_-intoxicated rats. Values are expressed as mean ± SEM

	Tailed DNA (%)	Tail length (μm)	Tail intensity (%)	Tail moment (Unit)
Control	4.25 ± 0.31	1.67 ± 0.045	2.56 ± 0.046	4.75 ± 0.161
CCl_4_	9.25^a***^ ± 0.44	3.25 ^a***^ ± 0.1	3.7 ^a***^ ± 0.068	11.74 ^a***^ ± 0.324
DFE+ CCl_4_	6.83 ^b**^ ± 0.31	2.48 ^b***^ ± 0.11	2.87 ^b***^ ± 0.038	7.84 ^b***^ ± 0.24
DPE+ CCl_4_	6.84 ^b**^ ± 0.477	2.83 ^b*c*^ ± 0.08	2.83 ^b***^ ± 0.057	9.12 ^b**c**^ ± 0.084
Coffee + CCl_4_	7.25 ^b**^ ± 0.362	2.64 ^b**^ ± 0.176	2.73 ^b***^ ± 0.042	8.43 ^b***^ ± 0.22

***P < 0.001, **P < 0.01, *P < 0.05

^a^significantly different from normal control group; ^b^significantly different from CCl_4_-treated group; ^c^significantly different from DFE-treated group

### Effect of DFE and DPE on expression of collagen I and collagen III

**Fig. 4:**
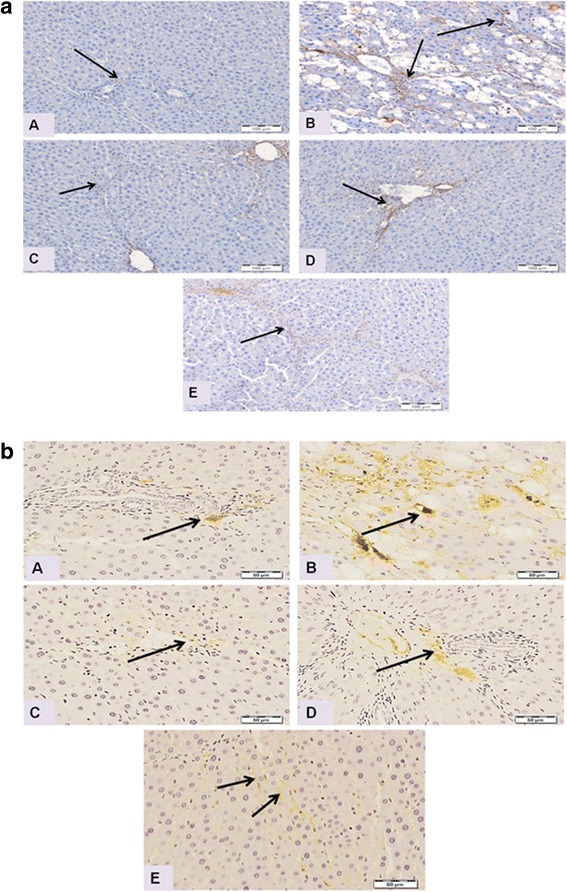
Effect of DFE, DPE and coffee on collagen deposition in CCl_4_-intoxicated rats. (a): Light microscopic photomicrographs of liver tissue immunostained with anti-collagen type I primary antibody (Scale bar = 100 μm). Panel **a** represents liver section from control group showing normal faint immunopositive areas that restricted to the portal areas (*arrow*). Panel **b** represents liver section of rat received CCl_4_ showing dense abnormal immune positivity of scattered patches near to the degenerated areas (*arrows*). Liver sections from rats treated with DFE (Panel **c**), DPE (Panel **d**) and coffee (Panel **e**) showing marked decrease of amount and density of abnormal fibrosis (*arrows*). The improvement is prominent in DFE-treated group. (b): Light microscopic photomicrographs of liver tissue immunostained with anti-collagen III primary antibody (Scale bar = 50 μm). Panel **a** represents liver section from control group showing normal immunopositive collagen distribution mainly in portal area (*arrow*), while Panel **b** represents liver section of rat received CCl_4_ in which strong wide abnormal irregularly distributed immune reactivity (*arrow*) was detected. Panels **c d** and **e** represent liver sections from rats treated with DFE DPE and coffee, respectively showing marked decrease of the intensity of the immune reactivity of hepatocytes surrounding portal areas especially in rats received DFE (*arrows*)

Immunostaining of collagen type I, the most common type of collagens in tissues, was performed to assess the degree of fibrosis in hepatic tissue (Fig. 4a). Liver sections from normal control group (Panel A) displayed normal faint immune positive areas that restricted to the portal areas. In contrast, liver sections from CCl_4_-inotxicated group showed dense abnormal immune positivity of scattered patches near to the degenerated areas (Panel B). Interestingly, treatment with DFE, DPE or coffee along with CCl_4_ showed marked decrease of the amount and density of abnormal fibrosis (Panels C to E, respectively). The improvement was notably prominent in rats treated with DFE. Immunohistochemical detection of collagen type III (Fig. 4b) supported these results where liver section of rat received CCl_4_ showed strong wide abnormal irregularly distributed immune reactivity (Panel B), whereas liver section from rats treated with DFE, DPE or coffee, revealed marked decrease of the intensity of the immune reactivity of hepatocytes surrounding portal areas especially in rats received DFE.

### Effect of DFE and DPE on expression of α-SMA in CCl_4_-intoxicated rats

**Fig. 5:**
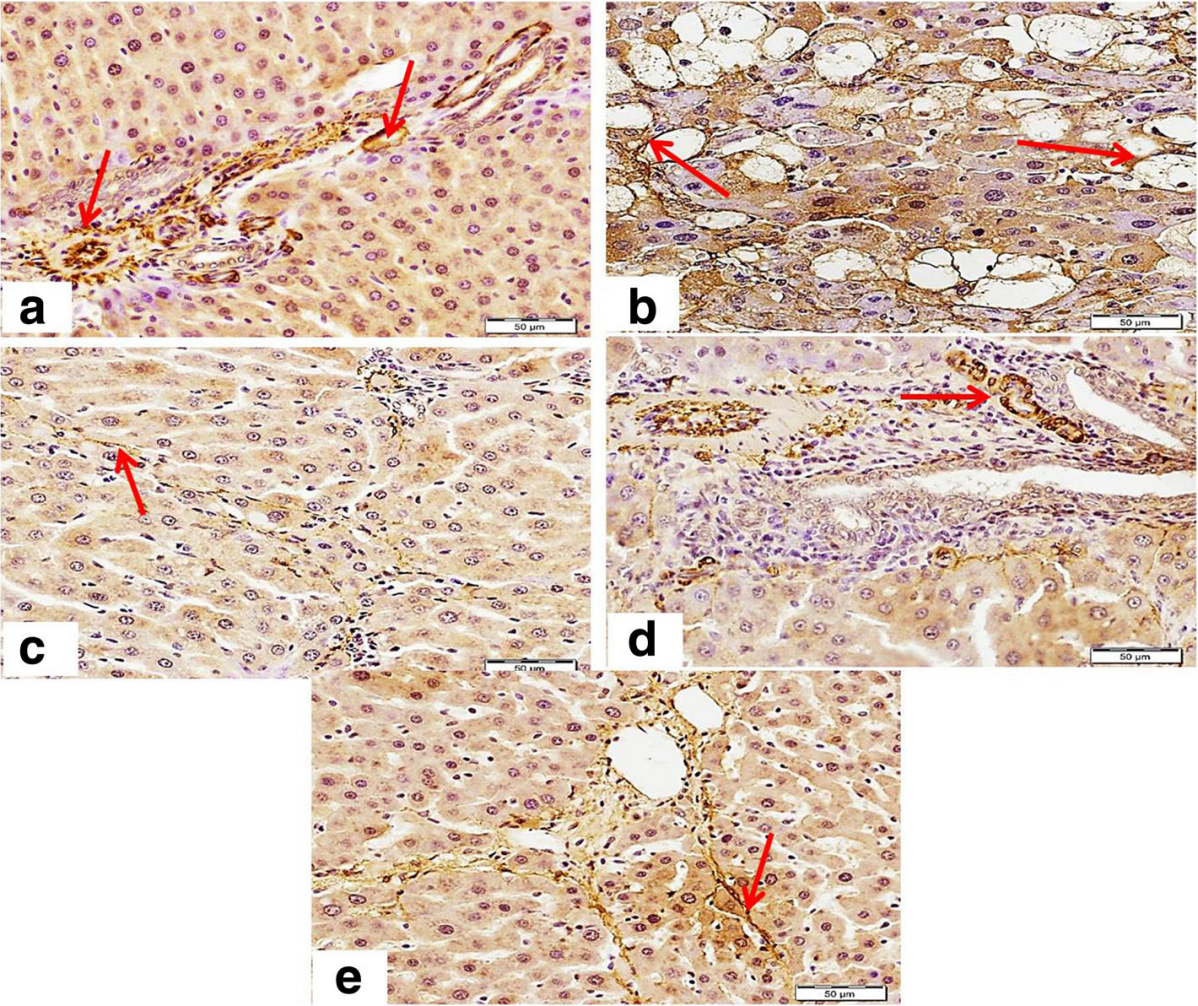
Light microscopic photomicrographs of liver tissue immunostained with primary anti α-SMA antibody (Scale bar = 50 μm). Panel **a** represents liver section from control rat showing normal positive immune reactivity of smooth muscle of the blood vessels of the portal area without immune staining positivity in between hepatocytes, while Panel **b** represents liver section of rat received CCl_4_ in which strong abnormal distributed immune reactivity, especially around degenerated hepatocytes, is prominent. Panels **c d** and **e** represent liver sections from rats received DFE, DPE and coffee, respectively, showing marked decrease of the immune reactivity outside portal areas especially in the group received DFE

Hepatic expression of α-SMA was detected as the key marker of HSCs activation (Fig. 5). HSCs are the major cellular source of collagen and their activation plays a key role on fibrogenesis. Liver section from control group (Panel A) revealed minimal staining of α-SMA in the blood vessels which restricted around the portal area without positive immunostaining between hepatocytes. In contrast, marked expression of α-SMA was observed in the CCl_4_-intoxicated rats particularly around degenerated hepatocytes, as shown by the intense brown staining (Panel B). Co-administration of DFE, DPE or coffee markedly attenuated this elevated expression (Panels C to E, respectively) where the immune reactivity is restricted to the vessels of the portal areas. The improvement was prominent with DFE.

### Effect of DFE or DPE on hepatic levels and expression of fibrolytic markers (MMP-9, TIMP-1 and TIMP-2) in CCl_4_-intoxicated rats

**Fig. 6:**
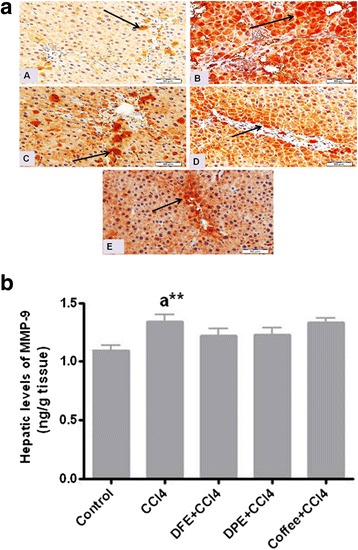
Effect of DFE and DPE on fibrolytic markers in CCl_4_-intoxicated rats. (a): Light microscopic photomicrographs of liver tissue immunostained with MMP-9 primary antibody (Scale bar = 50 μm). Panel **a** represents liver section from control group showing normal few immune positive cells’ cytoplasm (*arrow*) while the nuclei are not immunostained. Panel **b** represents liver section of rat intoxicated with CCl_4_ in which strong abnormal immune reactivity of most of hepatocytes cytoplasm and nuclei. Panels **c d** and **e** represent liver sections from rats treated with DFE, DPE and coffee, respectively revealed, many hepatocytes with strong positive immune reactivity in both cell’ cytoplasm and nuclei especially with coffee-treated group (*arrows*). (b): Effect of DFE, DPE and coffee on hepatic levels of MMP-9 in CCl_4_-intoxicated rats. Values are expressed as mean ± SEM. a: significantly different from normal control group. ** *P* < 0.01

**Fig. 7:**
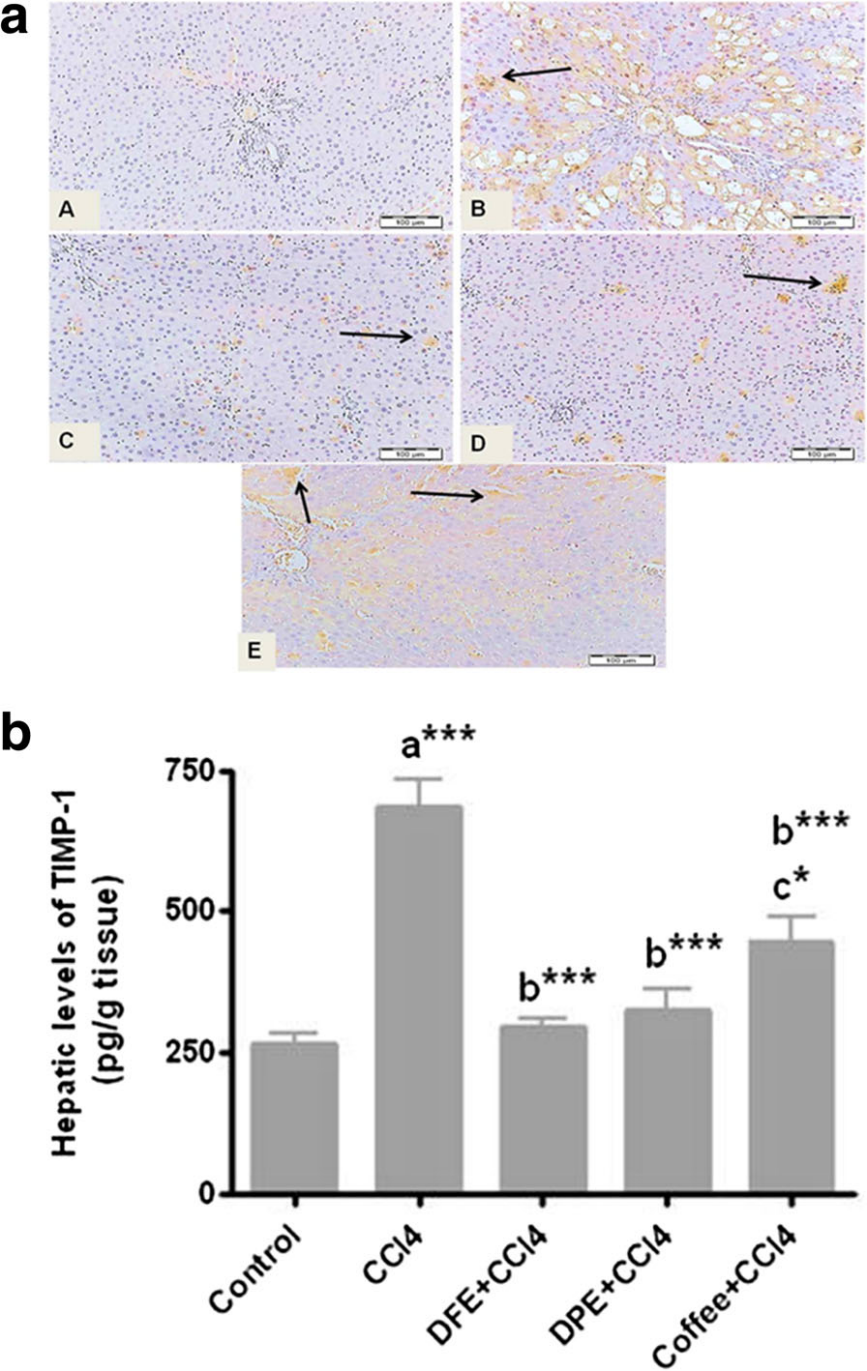
Effects of DFE and DPE on fibrolytic markers in CCl_4_-intoxicated rats. (a): Light microscopic photomicrographs of liver tissue immunostained with anti TIMP-2 primary antibody (Scale bar = 100 μm). Panel **a** represents liver section from control rat showing normal absence of immune positivity. Panel **b** represents liver section of rat intoxicated with CCl_4_ in which there are focal strong wide areas of abnormal immune reactivity of most of hepatic tissue. Panels **c d** and **e** represents liver sections from rats treated with DFE, DPE and coffee, respectively, revealed a decrease of immunostained areas which appeared weak in reaction. (b): Effect of DFE, DPE and coffee on hepatic levels of TIMP-1 in CCl_4_-intoxicated rats. Values are expressed as mean ± SEM. a: significantly different from normal control group; b: significantly different from CCl_4_-treated group; c: significantly different from DFE-treated rats. *** *P* < 0.001, * *P* < 0.05

Figure 6b showed that hepatic levels of MMP-9 were significantly elevated in CCl_4_-intoxicated rats compared to normal control group (*P* < 0.01). Concomitant treatment with DFE, DPE and coffee didn’t modify the MMP-9 levels compared to CCl_4_-intoxicated rats. These results were confirmed by the Immunohistochemical detection of MMP-9 in liver tissue (Fig. 6a) where rats received CCl_4_ exhibited strong abnormal immune reactivity of most of hepatocytes’ cytoplasm and nuclei (Panel B). Also, rats concomitantly received DFE, DPE or coffee revealed many hepatocytes with strong positive immune reactivity for MMP-9 (Panels C to E). Concerning TIMP-1 and -2, our results showed a marked increase in the hepatic levels of TIMP-1 in CCl_4_-treated rats compared to normal control (*P* < 0.001) (Fig. 7b). In addition, positive immune reactivity of TIMP-2 was observed in liver sections from CCl_4_-intoxicated rats (Fig. 7a, Panel B). The hepatic levels of TIMP-1 and immunoreactivity of TIMP-2 were significantly mitigated by treatment with DFE, DPE and coffee compared to CCl_4_-intoxicated rats (*P* < 0.001). The hepatic levels of TIMP-1 were significantly alleviated in DFE-treated rats compared to coffee-treated group (*P* < 0.05), while no significant differences were observed in DPE compared to either DFE- or coffee-treated group. The TIMP-2 expression detected by immunostaining revealed lower immune reactivity in both DFE- and DPE-treated groups (Fig. 7a, Panels C and D) compared to coffee (Panel E).

### Effect of DFE or DPE on inflammatory makers in CCl_4_-intoxicated rats

#### The effect on the expression of COX-2

**Fig. 8:**
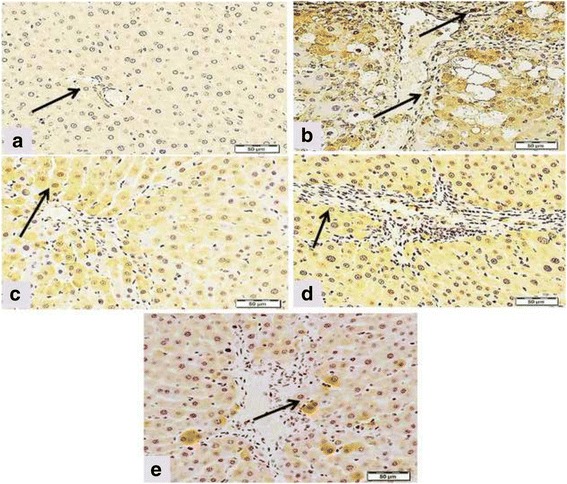
Light microscopic photomicrographs of liver tissue immunostained with primary anti COX-2 antibody (Scale bar = 50 μm). Panel **a** represents liver section from control rat showing very faint or weak immune-reactivity of hepatocytes, while panel **b** represents liver section of rat received CCl_4_ in which strong abnormal irregularly distributed immune reactivity was observed, especially between degenerated hepatocytes’ cytoplasm and nuclei (*arrow*). Many nuclei are also positively stained (*arrow*). Panels **c d** and **e** represent liver sections from rats treated with DFE, DPE and coffee, respectively showing marked decrease of the intensity of the immune reactivity of hepatocytes surrounding portal areas especially in rats treated with DFE (*arrows*)

The expression of the proinflammatory enzyme, COX-2 was estimated using immunohistochemical staining (Fig. 8). Liver section from normal control group showed minimal immunostaining for COX-2 (Panel A); while the section from CCl_4−_intoxicated rat revealed enhanced expression of this enzyme as shown by the intense brown staining (Panel B). Rats received CCl_4_ showed abnormal irregularly distributed immune reactivity of COX-2, especially between degenerated hepatocytes’ cytoplasm and nuclei and many nuclei are also positively stained. Concomitant treatment with DFE, DPE and coffee attenuated this elevation to a large extent (Panels C to E, respectively), where liver sections showed marked decrease of the intensity of the hepatocytes’ immune reaction surrounding portal areas especially in rats treated with DFE.

#### The effect on the expression of NF-κB (p65)

**Fig. 9:**
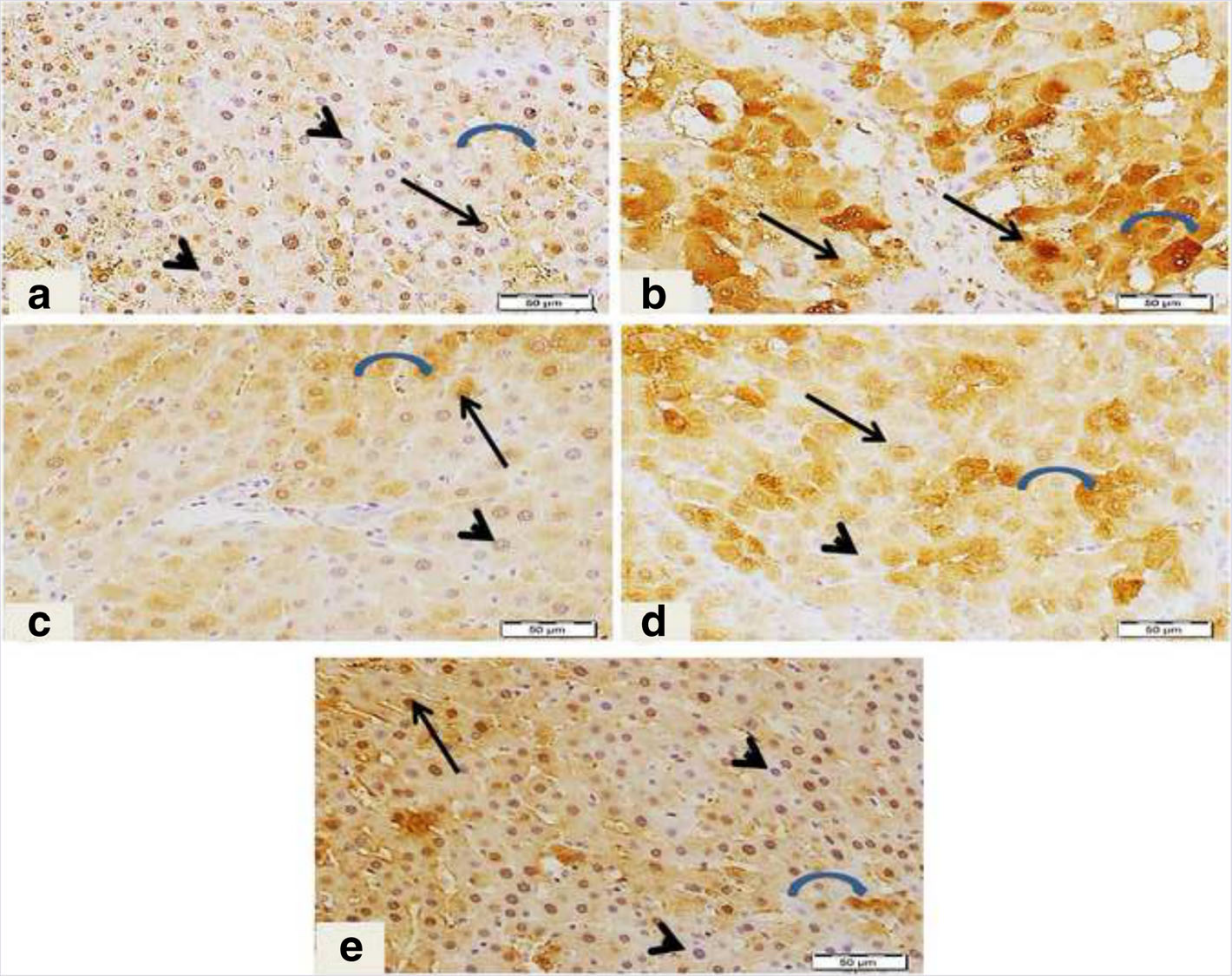
Light microscopic photomicrographs of liver tissue immunostained with anti NF-κB p65 primary antibody (Scale bar = 50 μm). Panel **a** represents liver section from control rat showing normal cytoplasmic distribution (*curved arrow*) with negative immune reactivity in most of nuclei (*arrowheads*). Only few weak immune positive nuclei (*arrows*) were observed. Panel **b** represents liver section of rat intoxicated with CCl_4_ in which there are focal strong abnormal immune reactivity of most of hepatocytes nuclei (*arrows*) and deeply stained cytoplasm (*curved arrows*). Panels **c d** and **e** represent liver sections from rat treated with DFE, DPE and coffee, respectively, revealing decreased number of immunostained nuclei of hepatocytes (*arrows*) where most of nuclei were negatively stained (*arrowheads*) particularly in DFE-and coffee-treated groups

NF-κB was assessed by detecting the activated subunit p65 in liver tissues (Fig. 9). Control rats showed minimal nuclear immunostaining for NF-κB p65 with normal cytoplasmic distribution (Panel A). CCl_4_ induced an increase in the p65 expression and activation in the liver tissues, which was evident from the intense staining of cytoplasm and focal strong abnormal immune reactivity of most of hepatocytes’ nuclei (Panel B). However, co-treatment with DFE, DPE and coffee significantly decreased the expression of NF-κB p65 (Panels C to E, respectively). Liver sections from rats received DFE, DPE or coffee revealed decreased number of immunostained nuclei of hepatocytes reflecting reduced nuclear translocation and suppression of activation particularly in rats treated with DFE and coffee.

#### The effect on the expression of HO-1

**Fig. 10:**
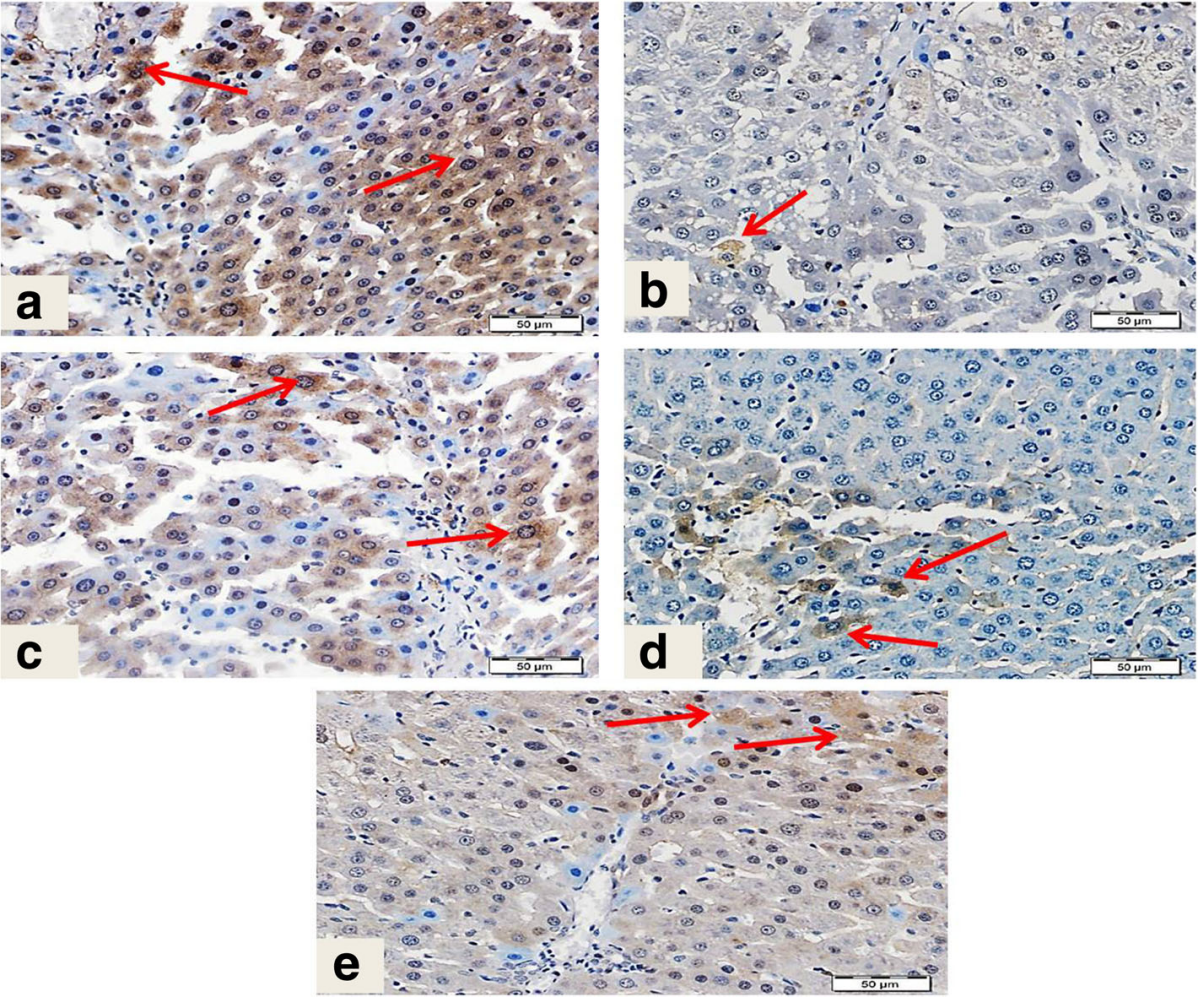
Light microscopic photomicrographs of liver tissue immunostained with anti-HO-1 antibody (Scale bar = 50 μm). Panel **a** represents liver section from control rat showing normal strong immune reaction of most of hepatocytes’ cytoplasm (*arrows*), while panel **b** represents liver section of rat received CCl_4_ with few hepatocytes showing weak immune reaction (*arrow*). Liver sections from rats treated with DFE (Panel **c**) and coffee (Panel **e**) showing patches of hepatocytes with moderate immune reaction (*arrows*). Panel D represents liver sections from rats treated with DPE showing mild weak immune positivity of few hepatocytes (*arrows*)

The expression of HO-1, an antioxidant and anti-inflammatory enzyme, was shown in Fig. 10. Normal control rat showed strong immune reaction of most of hepatocytes’ cytoplasm (Panel A), while CCl_4_-intoxicated rat’s liver revealed few hepatocytes with weak immune reaction (Panel B). Concurrent treatment with DFE (Panel C) and coffee (Panel E) showed patches of hepatocytes with moderate immune reaction, while, treatment with DPE showed less prominent improvement where mild weak immune positivity of few hepatocytes were observed (Panel D).

## Discussion

Carbon tetrachloride (CCl_4_) intoxication is a frequently used model of liver injury. CCl_4_ is activated by hepatic microsomal cytochromes to form the trichloromethyl (CCl3^•^) and trichloromethylperoxyl (OOCCl3^•^) radicals which are highly unstable and immediately activate oxidation of polyunsaturated fatty acids of hepatocellular membrane leading to its damage and increased permeability [62]. Another possibility is the covalent binding of CCl3^•^ to the hepatic microsomal lipids and proteins that damage the integrity of the structure and function of hepatic cell membranes [62] leading to leakage of liver enzymes into circulation and their elevation in serum (Fig. 1). Liver damage was also confirmed in the present study by the elevated levels of total and conjugated bilirubin that reflect the liver’s inability to take up, process, and secrete bilirubin into the bile. Recently, we reported the hepatoprotective effects of both DFE and DPE in CCl_4_-induced fibrosis in rats [38] via reducing oxidative stress, inactivation of HSCs, and reducing levels of inflammatory and angiogenic markers. In this study we focused on the following mechanisms: i-suppression of genotoxicity, ii- the fibrolytic potential of both extracts via modulating the expression of MMPs and TIMPs, and iii-downregulation of NF-κB pathway and/or upregulation of HO-1.

Cellular lipids are easily attacked by free radicals produced from the metabolism of CCl_4_ that initiates a chain reaction to cause lipid peroxidation and cell membrane damage [62]. Protein is also susceptible to damage by CCl_4_-induced free radicals. AOPPs are dityrosine-containing cross-linked protein products formed during oxidative stress and are considered to be reliable markers for estimating the degree of oxidant-mediated protein damage [63]. In our study, CCl_4_ resulted in increased hepatic levels of MDA and plasma levels of AOPPs indicating lipid peroxidation and the oxidative protein damage, respectively. In addition, hepatic CAT activity was reduced reflecting the consumption of this antioxidant enzyme. Moreover, we previously demonstrated that CCl_4_ caused depletion of GSH, SOD and GPx confirming the oxidative stress in liver tissue [38]. Our previous and present work reported that both DFE and DPE exhibited potent protective effects against CCl_4_-mediated oxidative damage via decreasing hepatic levels of MDA and increasing the levels and activities of GSH, CAT, SOD and GPx. In addition, the present work also demonstrated that DFE and DPE alleviated protein oxidation as evidenced by reduced plasma levels of AOPPs.

Like other macromolecules such as lipids and proteins, nucleic acids are also attacked by free radicals to cause oxidative DNA damage. DNA strand breaks and formation of 8-OHdG are abundant forms of oxidative damage induced by highly reactive free radicals [13, 14]. Even when the levels of these radicals are too low to cause such lethal damage, chronic exposure to cells can lead to accumulation of 8-OHdG in DNA. 8-OHdG is generated by oxidation of C8 of guanine and forms pairs with undesirable adenine residue in addition to authentic cytosine residue in DNA, thereby leading to a greatly increased frequency of G:C to T:A transversion mutation and DNA damage [64]. Moreover, and according to Marnett [65], the product of lipid peroxidation react with DNA to form adducts MIG, the mutagenic adduct of deoxyguanosine. Alkreathy et al. [66] demonstrated that CCl_4_ degrades the DNA of liver tissue of rats by generating free radicals. In the present study, chronic exposure to CCl_4_ lead to a significant rise in the hepatic levels of 8-OHdG in addition to DNA fragmentation indicated by significant increase in tailed DNA %, tail length, tail intensity and tail moments which is probably associated with increased production of reactive oxygen species. Various forms of oxidative damage induced by CCl_4_ in liver may be prevented by antioxidants supplementation which represents a rationale for using them in the treatment of liver disorders. In this regard, the protective effects of DFE and DPE against CCl_4_-induced genotoxicity were evaluated in the current work. We found that hepatic levels of 8-OHdG and all indices of DNA fragmentation (tailed DNA %, tail length, tail intensity and tail moments) were significantly lower in DFE- and DPE- treated rats supporting the protective effects of both extracts against genotoxicity. These protective effects could be attributed to the antioxidant potential of DFE and DPE that is believed to be due to the wide range of polyphenolic compounds (p-coumaric, ferulic, sinapic acids, flavonoids, anthocyanins, phenolic acids and procyanidins), and trace elements (selenium, copper, zinc and manganese), in addition to vitamin C present in the date palm fruit flesh [23, 27] and pits [37]. The antioxidant potential of DFE and DPE may be also related to their ability, particularly DFE, to preserve the expression of the antioxidant enzyme, HO-1, in liver cells (Fig. 10). Our work is the first in vivo study investigating the protective role of dates fruit extract against oxidative DNA damage. Similar to our results, the in vitro study performed by Vayalil [22] proved the antioxidant and antimutagenic activity of the aqueous date palm fruit extract resulting from the inhibition of both lipid and protein oxidation and also by the aptitude of this extract to scavenge superoxide and hydroxyl radicals. Our results concerning coffee, as a reference antifibrotic agent, supported the previous studies reporting that coffee intake is associated with lower levels of oxidative DNA damage and reduced levels of 8-OHdG [49–51]. Various components of coffee that have been related to such a favorable effect include caffeine, coffee oils kahweol, cafestol and antioxidant substances.

Fibrogenesis is mainly characterized by the activation of HSCs with increased production of ECM particularly collagens and the secretion of α-SMA; the hall mark for HSC activation [1]. Our results revealed significantly higher expression of collagen I, collagen III and α-SMA in the liver of CCl_4_-intoxicated rats indicating fibrogenesis. On the other hand, treatment with DFE and DPE successfully alleviated the fibrotic changes in rat’s liver as indicated by the reduced expression of α-SMA, collagen I and collagen III which confirm our previous results reporting the ability of DFE and DPE to reduce collagen deposition, hydroxyproline and TGF-β1, the most potent fibrotic factor, in hepatic cells [38]. Among several types of cells and cytokines involved in HSCs activation, the transcription factor NF-κB plays a crucial role [67]. DNA binding activity of NF-κB is demonstrated in activated but not in quiescent HSCs and activation of HSCs is associated with the nuclear translocation of NF-κB. The current results suggest that the strong immunostaining of NF-κB p65 (Fig. 9, panel B) may play a pivotal role in HSCs activation. Moreover, the current study suggest that the ability of both DFE and DPE to inhibit HSCs activation and collagen accumulation is partly due to blocking NF-κB activation indicated in the present study by decreased NF-κB p65 immunoreactivity (Fig. 9, panels C and D). We previously presented additional mechanism of the inhibitory effect of DFE and DPE on HSCs activation via reducing the levels of TGF-β1, TNF-α, IL-6 and IL-1β, the most important cytokines involved in HSCs activation [38].

To further investigate the antifibrotic mechanisms of DFE and DPE in hepatic cells, their ability to induce collagen degradation was elucidated in the current study. In healthy liver, MMPs and TIMPs play an important role in inducing and preventing the degradation of the ECM, respectively. The imbalance between MMPs and TIMPs is considered a pivotal mechanism of ECM deposition and liver fibrogenesis [5]. Among different MMPs and TIMPs, MMP-9 is known as the regulator for the breakdown of ECM, while TIMP-1 and -2 exhibit anti-fibrolytic and growth-stimulated activities [68]. In this study, CCl_4_-intoxicated rats revealed significantly higher levels and expression of MMP-9 which is consistent with the previous studies conducted on chronic models of CCl_4_-induced liver fibrosis [69–71]. Moreover, MMP-9 is produced in CCl_4_-induced liver injury after a single injection [72] and in hepatitis C virus-induced cirrhosis [73]; however, its activity was not related to the degree of fibrosis [74]. In this regard, the levels and expression of MMP-9 in our study were increased in CCl_4_-intoxicated rats’ liver, possibly due to HSC activation [3]. It has been demonstrated that the antifibrotic efficacy is associated with a decreased hepatic level of MMP-9 [70]. However, in our study, no significant change in the levels and expression of MMP-9 was observed by treatment with DFE, DPE or coffee compared to CCl_4_-intoxicated rats suggesting the antifibrotic effect may not be related to modulation of MMP-9 activity.

TIMP-1 and TIMP-2 are capable of inhibiting the activities of all known MMPs and, in consistent with our results, their expressions are increased in CCl_4_-induced liver fibrosis [69, 70, 74]. Activation of HSCs in culture and in vivo is accompanied by increased expression and secretion of TIMP-1 and TIMP-2 [74, 75]. Therefore in the presence of a hepatotoxic agent, HSCs are activated, and the TIMP-1 and -2 are upregulated, leading to fibrogenesis. On the other hand, fibrosis resolution has been correlated with a diminution of TIMP-1 and TIMP-2 [75, 76], therefore, TIMP-1 and TIMP-2 represent important therapeutic target of the antifibrotic strategies for chronic liver disease [69, 76]. Our results revealed significant reduction in the hepatic levels of TIMP-1 and expression of TIMP-2 in rats treated with DFE or DPE which imply a new and significant antifibrotic mechanism for DFE or DPE via fibrolytic activity. This significant downregulation in the hepatic level of TIMP-1 and TIMP-2 by DFE and DPE might be due to the following: First, the inhibition of HSCs activity, as indicated by lowered expression of α-SMA (Fig. 5, panel C and D); second, the diminished hepatic levels of TGF-β1 by DFE and DPE [38]. This inhibitory effect on TGF-β1 is important because TGF-β1 plays a key role in modulating the levels of ECM via inhibiting MMPs, while simultaneously up-regulates expression of TIMPs [77] leading to failure of ECM degradation and accumulation of collagen in hepatic tissue. The third explanation of the fibrolytic activity of DFE and DPE is their inhibitory effect on the secretion of inflammatory cytokines, TNF-α, IL-6 and IL-1β [38] which are known to upregulate the TIMP-1 levels in rat and human hepatocytes [78]. Our suggestion is supported by the study of Roderfeld et al. [72] who demonstrated that cytokine blockade inhibits the expression of TIMP-1 and up-regulates the expression of MMP-9. In the current study, immunohistochemical examinations showed that livers from DFE- and DPE-treated rats exhibited significant reduction in collagens type I and type III reactivity (Fig. 4) suggesting the beneficial role of DFE and DPE in the removal of deposited collagen. This enhanced collagenolytic activity could be strongly associated to the decreased TIMP-1 and TIMP-2 levels by both extracts. In conclusion, enhancement of matrix degradation might be involved in the antifibrotic effects of DFE and DPE.

The proinflammatory mediators, TNF-α, IL-6 and IL-1β can promote fibrosis via different mechanisms. Recently, we reported the anti-inflammatory effects of DFE and DPE in CCl_4_-iduced liver fibrosis via reducing the aforementioned mediators [38]. However, the data concerning anti-inflammatory effect of dates in liver toxicity is still poor and inadequate. In the current study, we aimed to investigate whether the anti-inflammatory effects of DFE and DPE are mediated through the modulation of NF-κB and/or HO-1 pathways.

NF-κB provides an important mechanism of the inflammatory response in liver fibrosis. Activation of NF-κB leads to production of the proinflammatory cytokines, followed by activation of other inflammatory mediators, such as COX-2 and iNOS [9]. In the current study, CCl_4_-injured liver demonstrated strong immunostaining of NF-κB p65 subunit that is located in nucleus. Cytoplasmic NF-κB activation and its subsequent nuclear translocation in CCl_4_-injured livers are suggested to be induced by CCl_4_-generated free radicals [79]. NF-κB was also activated during transdifferentiation of HSCs into myofibroblasts like cells [1]. Importantly, the accumulation and nuclear translocation of the NF-κB p65 subunit in the liver cells of CCl_4_-intoxicated rats was significantly attenuated by treatment with DFE or DPE suggesting their anti-inflammatory activity through suppression of NF-κB activation. The inhibitory effects of DFE and DPE on NF-κB p65 may be attributed to their role in HSCs inactivation or may be related to their polyphenols, flavonoids and anthocyanins contents which are documented to suppress COX-2 expression and inhibit NF-κB translocation in previous studies [80]. Moreover, the inhibitory effect of DFE and DPE on NF-κB could be further explained by their ability, particularly DFE, to preserve HO-1 enzymatic activity as demonstrated in the current study in Fig. 10 (panels C and D). Yang et al. [81] demonstrated a potential mechanism that HO-1 prevents the expression of NF-κB in fibrotic liver tissue. In addition, Xue et al. [18] suggest that high levels of HO-1 expression may decrease NF-κB expression to lessen the inflammatory reaction at 6 h after reperfusion in cirrhotic rats. Concerning coffee, our results supports previous data reported the inhibitory effect of coffee on NF-κB in vitro and in vivo [52–54]. The coffee-induced NF-κB inhibition activity was attributed mainly to polyphenolics and melanoidins, two major coffee chemical groups [53, 54]. The upregulation of HO-1 enzyme activity as demonstrated in the current study (Fig. 10, panel E) may further explain the role of coffee in inhibiting NF-κB activation.

The cyclooxygenase (COX) pathway is responsible for the conversion of arachidonic acid into prostaglandins; the most widely recognized mediators of inflammation. COX-1 and COX-2 are two isoforms of the COX enzyme. Accumulated evidences indicate that the COX-2 pathway has been implicated in liver inflammation, matrix remodelling, fibrosis progress and development of hepatocellular carcinoma [82, 83]. Previous data also documented increased expression and enhanced formation of COX-2 in CCl_4_-induced liver injury [82–84] which is consistent with our results where strong immunoreactivity of COX-2 was detected in liver of CCl_4_-treated rats. NF-κB is involved in the regulation of COX-2 gene expression by binding to its promoter regions and enhancing the COX-2 transcription [85]. The enhanced activation and translocation of NF-κB p65 in the present study may explain the increased expression of COX-2 in CCl_4_-induced liver injury. Importantly, COX-2 inhibition was beneficial in CCl_4_-induced fibrosis as it reduces cell growth and triggers apoptosis in HSCs and exerts antifibrogenic actions [82–84]. It is obvious from our study that both DFE and DPE down-regulated the activation of NF-κB and simultaneously suppressed COX-2 expression, suggesting the inhibition of this proinflammatory pathway may be a potential strategy for prevention of inflammatory liver injury and fibrogenesis. In agreement with our results, Zhang et al. [86] reported that Ajwa, a variety of date palm *Phoenix dactylifera L*. is very active as anti-inflammatory and inhibited both COX-1, COX-2 activity.

The HO-1 plays an important role in various pathophysiological conditions owing to its antioxidative, anti-inflammatory, antiapoptosis, and potent cytoprotective properties [16–20]. Thus, pharmacological modulation of the HO-1 system may represent an effective and cooperative strategy to mitigate liver injury. In the current study, CCl_4_ induced downregulation of HO-1 expression (Fig. 10, Panel B) which may contribute to oxidative stress, inflammatory response and fibrogenesis in hepatic cells. Co-administration of DFE, DPE or coffee significantly ameliorated this reduction, particularly with DFE and coffee suggesting a new mechanism for their antifibrotic action. Previous reports demonstrated that HO-1 induction by coffee is attributed to polyphenolic constituents, the most abundant form being chlorogenic acid and caffeic acid. In addition, it has been demonstrated that caffeic acid and kahweol, polyphenolic and diterpine constituents of coffee, drastically activated the HO-1 gene with elevated induction of HO-1 leading to the control of intracellular levels of ROS [55, 56]. Except for the current study, no data are available concerning the role of DFE or DPE on the induction HO-1. Future studies are recommended to evaluate which constituents of dates are involved in the HO-1 induction in hepatic cells.

## Conclusion

In summary, data from our study indicated that the potential of DFE and DPE to suppress TIMP-1 and -2 expressions in liver helps to explain, at least in part, the fibrolytic effect of dates enhancing the degradation of ECM and minimizing the collagen deposition. In addition, our results demonstrated that, the anti-inflammatory potential of dates is strongly related to the inhibition of NF-κB translocation, reduced expression of COX-2 and restoration of HO-1 activity. The antifibrotic effects of dates could be also associated with reduced DNA oxidative damage possibly through the elimination of CCl_4_-generated free radicals. The present study revealed a clinical potential of date fruits either flesh or pits for therapeutic antifibrotic applications.

## Additional file

Additional file 1: Effect of DFE, DPE and coffee on liver function markers, hepatic levels of 8-OHdG, MMP-9 and TIMP-1 in CCl_4_-intoxicated rats a: significantly different from normal control group; b: significantly different from CCl_4_-treated group. Values are expressed as mean ± SEM. *** P < 0.001, ** P < 0.01. ALT: alanine aminotransferase; AST: aspartate aminotransferase. (XLS 23 kb)

## Abbreviations

8-OHdG: 8-hydroxy-2’- deoxyguanosine
ALT: Alanine aminotransferase
ANOVA: One-way analysis of variance
AOPPs: Advanced oxidation protein products
AST: Aspartate aminotransferase
CAT: Catalase
CCl_4_: Carbon tetrachloride
COX-2: Cyclooxygenase-2
DFE: Date flesh extract
DPE: Date pits extract
ECM: Extracellular matrix
ELISA: Enzyme-linked immunosorbent assay
GPx: Glutathione peroxidise
GSH: Glutathione
HO-1: Heme oxygenase-1
HSCs: Hepatic stellate cells
IL-1β: Interleukin-1 beta
IL-6: Interleukin-6
iNOS: Inducible nitric oxide synthase
MDA: Malondialdehyde
MMPs: Matrix metalloproteinases
NF-κB: Nuclear factor- kappa B
NMP: Normal melting point agarose
ROS: Reactive oxygen species
SOD: Superoxide dismutase
TBA: Thiobarbituric acid
TBARS: Thiobarbituric acid reactive substances
TBE: Trisborate- EDTA
TCA: Trichloroacetic acid
TGF-β1: Transforming growth factor-beta 1
TI: Tail intensity
TIMPs: Tissue inhibitors of metalloproteinases
TL: Tail length
TMOM: Tail moment
TNF-α: Tumor necrosis factor-alpha
α-SMA: Alpha-smooth muscle actin
